# Genome-Wide Identification, Expression, and Protein Analysis of CKX and IPT Gene Families in Radish (*Raphanus sativus* L.) Reveal Their Involvement in Clubroot Resistance

**DOI:** 10.3390/ijms25168974

**Published:** 2024-08-17

**Authors:** Haohui Yang, Xiaochun Wei, Weiwei Lei, Henan Su, Yanyan Zhao, Yuxiang Yuan, Xiaowei Zhang, Xixiang Li

**Affiliations:** 1 Institute of Vegetables, Henan Academy of Agricultural Sciences, Zhengzhou 450002, China; yanghaohui127@163.com (H.Y.); jweixiaochun@126.com (X.W.); 18810835083@163.com (H.S.); zhaoyanyan9621@163.com (Y.Z.); yuxiangyuan126@126.com (Y.Y.); 2 State Key Laboratory of Vegetable Biobreeding, Key Laboratory of Biology and Genetic Improvement of Horticultural Crops, Ministry of Agriculture and Rural Affairs, Institute of Vegetables and Flowers, Chinese Academy of Agricultural Sciences, Beijing 100081, China; 3 Station for Popularizing Agricultural Technique of Changping District, Beijing 102200, China

**Keywords:** *Raphanus sativus*, *RsIPT*, *RsCKX*, clubroot resistance, expression profile, 3D structure, interaction network

## Abstract

Cytokinins (CKs) are a group of phytohormones that are involved in plant growth, development, and disease resistance. The isopentenyl transferase (IPT) and cytokinin oxidase/dehydrogenase (CKX) families comprise key enzymes controlling CK biosynthesis and degradation. However, an integrated analysis of these two gene families in radish has not yet been explored. In this study, 13 *RsIPT* and 12 *RsCKX* genes were identified and characterized, most of which had four copies in *Brassica napus* and two copies in radish and other diploid *Brassica* species. Promoter analysis indicated that the genes contained at least one phytohormone or defense and stress responsiveness cis-acting element. *RsIPTs* and *RsCKXs* were expanded through segmental duplication. Moreover, strong purifying selection drove the evolution of the two gene families. The expression of the *RsIPT* and *RsCKX* genes distinctly showed diversity in different tissues and developmental stages of the root. Expression profiling showed that *RsCKX1-1/1-2/1-3* was significantly upregulated in club-resistant materials during primary infection, suggesting their vital function in clubroot resistance. The interaction network of CKX proteins with similar 3D structures also reflected the important role of *RsCKX* genes in disease resistance. This study provides a foundation for further functional study on the *IPT* and *CKX* genes for clubroot resistance improvement in *Raphanus*.

## 1. Introduction

Radish (*Raphanus sativus* L. 2n = 18), belonging to the Brassicaceae, is an important vegetable and oil crop cultivated widely around the world. Clubroot disease, which is caused by biotrophic protist *Plasmodiophora brassicae* Woronin, leads to serious losses in radish, Chinese cabbage (*Brassica rapa* L. ssp. *pekinensis* Lour., 2n = 20), and other Cruciferous crops and has gradually evolved into a catastrophic disease worldwide [1,2,3]. *Plasmodiophora brassicae* has a complex life cycle, while the infection process comprises two distinct phases. The two phases occur in different parts of plant roots and have different infection routes. The primary phase starts with infection by primary zoospores, whose resting spores germinate in root hair and epidermal cells [4]. Then, the primary zoospores form primary plasmodia, further split into zoosporangia, and produce secondary zoospores in epidermal cells. A sign of the secondary phase is that the secondary zoospores infect cortical tissue. Subsequently, secondary zoospores develop into secondary plasmodia and then release resting spores into the soil. It is difficult to manage and control clubroot disease due to the persistent vitality and diverse transmission of *P. brassicae* [5,6].

Cytokinins (CKs) are essential phytohormones that are involved in various biological processes related to plant growth and development, such as cell division in roots and shoots, apical dominance, shoot or root branching, leaf development, bud growth, seed germination, photosynthesis, senescence, and nutrient metabolism [7]. Furthermore, CKs participate in the response to multiple stress factors and have a significant role in plant immunity against microorganisms [8,9,10,11,12]. Thus far, CKs have been considered connected to disease symptoms and abnormal morphogenesis, such as the formation of nodules or galls, fasciation, senescence, and the generation of green islands [13,14]. Some studies have shown that CKs mediate plant susceptibility to gall-causing pathogens and biotrophic fungi [15,16,17,18].

Typically, a host plant has its own stable source–sink relationship; pathogen invasion can affect the reconfiguration of plant metabolism, such as by altering the hormone balance, leading to a new source–sink relationship [17,19]. Previous studies have shown that the alteration of plant root hormone homeostasis plays a prominent part in clubroot formation [20,21,22,23]. In particular, the alteration of CK homeostasis driven by *P. brassicae* induces cell division and hypertrophy by reconfiguring existing meristematic activity, resulting in root nodules [24,25,26]. In *Arabidopsis thaliana* L. (2n = 10), research has shown that the CK concentration increases in root galls after infection with *P. brassicae* [27,28,29]. Further research has shown that the CK concentration increases in the early phase but decreases in the later period of root hypertrophy in *Arabidopsis* [22,30]. In turnips (*Brassica rapa* subsp. *Rapa* L., 2n = 20) and rapeseed (*Brassica napus* L., 2n = 38), the CK content is higher in infected roots than in non-infected roots [23,31]. CK-depleted *Arabidopsis* plants are more resistant to clubroot disease [21,23]. Therefore, previous studies have clearly indicated that CKs are a key factor in plant resistance to *P. brassicae*.

Although multiple CK biosynthesis routes have been proposed, CK biosynthesis is mainly catalyzed by the isopentenyl transferase (IPT) family [8,32,33,34]. The synthesis of isopentenyladenine(ip)- and trans-zeatin(tz)-type CKs is controlled by ATP/ADP IPTs, and the synthesis of cis-zeatin-type CKs is controlled by tRNA IPTs. The degradation processes of CKs and their derivatives are mainly catalyzed by CK oxidase/dehydrogenases (CKXs).

*IPT* and *CKX* gene families have been analyzed in crucifer crops such as *Arabidopsis thaliana*, *Brassica rapa* L. (2n = 20), *Brassica oleracea* L. (2n = 18), and *Brassica napus* L. (2n = 38) [32,35,36,37,38,39]. In *Arabidopsis*, nine *IPT* members (*AtIPT1*–*AtIPT9*) and seven *CKX* members (*AtCKX1*–*AtCKX7*) have been identified [32,40]. In *B. rapa*, 13 *BrIPT* genes and 12 *BrCKX* genes have been identified [36]. In *B. oleracea*, 36 *CKX* genes have been identified [38]. In *B. napus*, a total of twenty-six *IPT* genes were identified [39]. Further function analysis has been carried out on these two family members in *Arabidopsis*. The overexpression of one *IPT* gene, *AtIPT4*, which has unique isopentenyltransferase activity for DMAPP: ATP/ADP in its purified form, led to CK-independent shoot formation on *Arabidopsis* calli [41]. The overexpression of *AtCKX* genes in transgenic tobacco and its own plants displays various degrees of reduction of cytokinin or CK-deficiency traits [35,42]. Compared to genes related to CK biosynthesis and degradation, which have been extensively studied in *Arabidopsis*, research on these genes has been less frequently reported in other crucifer crops. In particular, genome-wide identification of *IPT* and *CKX* gene family members has still not been reported in radish. It is unclear what role *IPT* and *CKX* genes play in the radish-*P. brassicae* interaction.

## 2. Results

### 2.1. Genome-Wide Identification and Distribution of CKX and IPT Genes in Radish

*CKX* and *IPT* genes were identified based on BLAST and hmmsearch among Cruciferous crops. A total of 13 IPT and 12 *CKX* genes were identified in radish. Based on homologous genes in *A. thaliana* (*AtCKX* and *AtIPT*), these genes were named the *RsIPT* and *RsCKX* genes. Furthermore, *IPT* and *CKX* genes were identified in other Cruciferous crops. Specifically, 9, 13, 13, 15, and 30 *IPT* genes and 7, 12, 12, 12, and 25 *CKX* genes were identified in *Arabidopsis thaliana* (2n = 10), *Brassica rapa* (2n = 20), *Brassica oleracea* (2n = 18), *Brassica nigra* L. (2n = 16), and *Brassica napus* (2n = 38), respectively (Table S1). The number of *CKX* and *IPT* genes showed obvious diversity among species with different ploidy and increased when ploidies doubled.

The physicochemical properties of the *IPT* and *CKX* genes were predicted (Table 1 and Table 2). The proteins of 13 *RsIPT* genes ranged from 113 (*RsIPT7-2*) to 499 (*RsIPT9-1*) amino acids in length, while 12 *RsCKX* genes ranged from 387 (*RsCKX1-2*) to 1033 (*RsCKX3-1*) amino acids in length. The relative molecular weight (MW) of the *RsIPT* genes varied from 12,356.3 Da (*RsIPT7-2*) to 56,375.9 Da (*RsIPT9-1*), while the MW of the *RsCKX* genes varied from 43,085.8 Da (*RsCKX1-2*) to 116,376 Da (*RsCKX3-1*). The PI of the *RsIPT* genes ranged from 5.28 (*RsIPT10*) to 10.35 (*RsIPT7-2*), while the PI of *RsCKX* ranged from 4.38 (*RsCKX7-2*) to 9.35 (*RsCKX1-2*). The predicted subcellular locations of *RsIPT* genes showed that most were in the chloroplast, and a few were located in the cytoskeleton, nucleus, and mitochondrion. Among the *RsCKX* genes, four were in the mitochondrion, two in the chloroplast, extracell, and cytoplasm, and one was in the vacuole and plasma membrane. The *RsCKX* genes showed more diversity in subcellular localization than the *RsIPT* genes. The paralogous genes, namely *RsIPT9-1/9-2*, *RsIPT7-1/7-2*, *RsCKX1-1/1-2/1-3*, and *RsCKX7-1/7-2*, were in the same subcellular location, while other paralogs were distributed in different locations.

**Figure 1:**
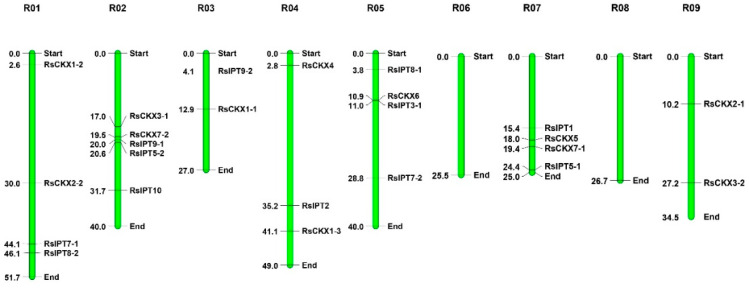
Chromosomal mapping of the *RsIPT* and *RsCKX* genes in radish. The green bars represent chromosomes. Values corresponding to the *RsIPT* and *RsCKX* genes represent the physical distance (Mb).

The *RsIPT* and *RsCKX* genes were mapped to radish chromosomes (Figure 1). *RsIPT3-2* was unmapped on a chromosome due to its location on scaffold 638. The overall distribution of the *RsIPT* and *RsCKX* genes on nine chromosomes was uneven. Chromosome 2 (R02) had the most *RsIPT* and *RsCKX* genes, followed by R01, R05, and R07. Specifically, five genes were located on R02, four genes were located on R01, R05, and R07, and three genes were located on R04. Both R03 and R09 had two genes. However, no *RsIPT* or *RsCKX* genes were identified on R06 and R08. No *RsIPT* genes were identified on R09.

### 2.2. Phylogenetic Analysis of RsIPT and RsCKX Gene Families

**Figure 2:**
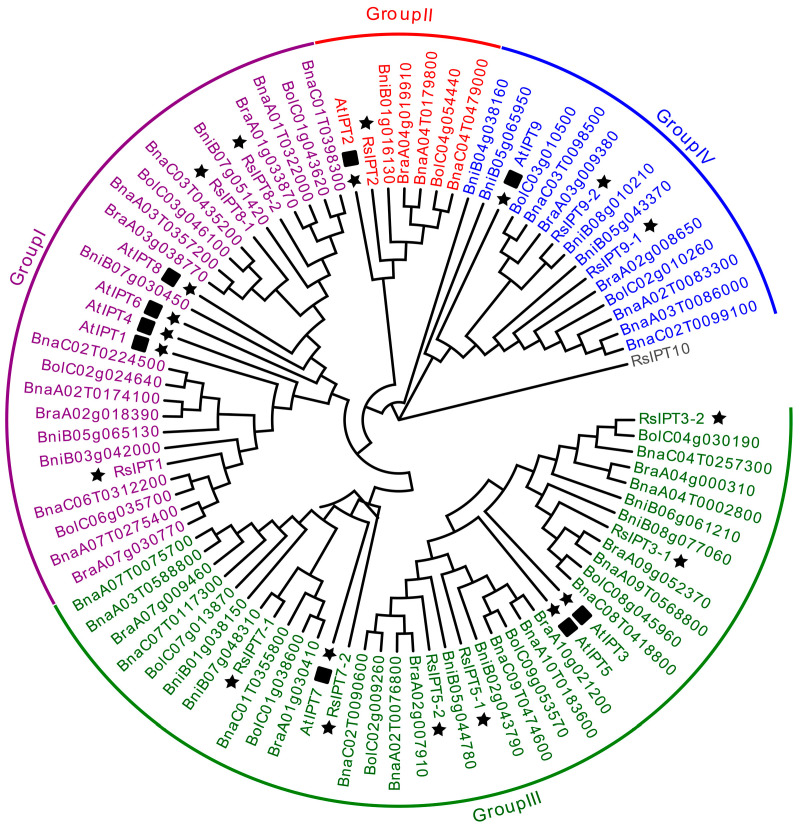
Phylogenetic tree of *IPT* genes from *Arabidopsis thaliana*, *Brassica rapa*, *B. oleracea*, *B. nigra*, *B. napus*, and *Raphanus sativus*. *IPT* genes are divided into four groups according to phylogenetic analysis. Different groups are indicated with corresponding colors. The gene *RsIPT10* which not belong to any group was presented by gray color. The *IPT* genes of *R. sativus* are highlighted by black stars, and the *IPT* genes of *A. thaliana* are highlighted by black stars and squares. The *A. thaliana*, *B. rapa*, *B. oleracea*, *B. nigra*, *B. napus*, and *R. sativus* genes are marked with “At”, “Bra”, “Bol”, “Bni”, “Bna”, and “Rs”, respectively.

**Figure 3:**
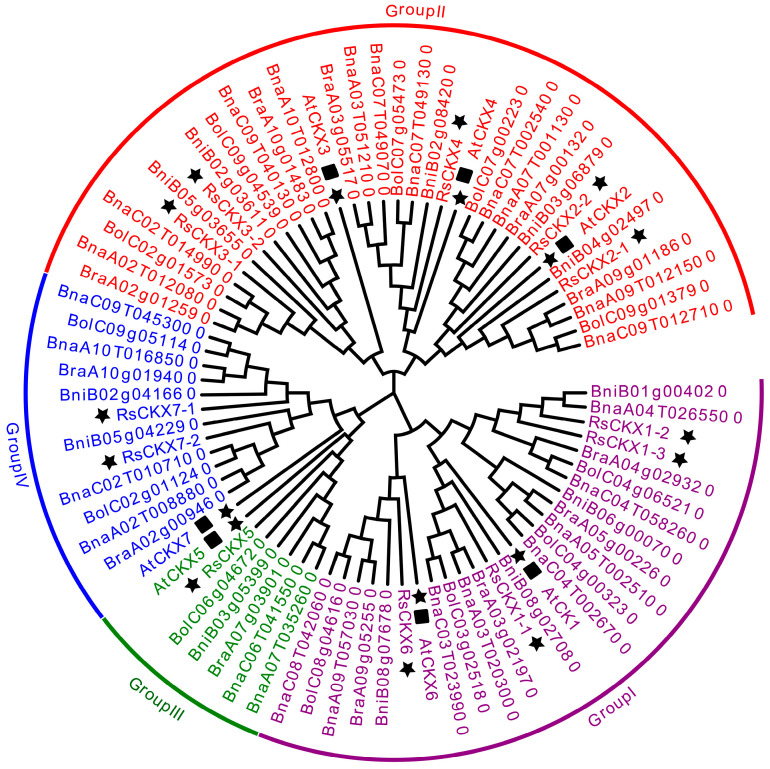
Phylogenetic tree of *CKX* genes from *Arabidopsis thaliana*, *Brassica rapa*, *B. oleracea*, *B. nigra*, *B. napus*, and *Raphanus sativus*. *CKX* genes are divided into four groups according to phylogenetic analysis. Different groups are indicated with corresponding colors. The *IPT* genes of *R. sativus* are highlighted by black stars, and the *IPT* genes of *A. thaliana* are highlighted by black stars and squares. The *A. thaliana*, *B. rapa*, *B. oleracea*, *B. nigra*, *B. napus*, and *R. sativus* genes are marked with “At”, “Bra”, “Bol”, “Bni”, “Bna”, and “Rs”, respectively.

To study the evolutionary relationships and identify the subfamilies in the *RsIPT* and *RsCKX* gene families, phylogenetic analysis of the *IPT* and *CKX* genes was performed in *A. thaliana*, *B. rapa*, *B. oleracea*, *B. nigra*, *B. napus*, and *R. sativus* using MEGA with protein sequence information. Based on the phylogenetic tree, the *RsIPT* genes were classified into four subgroups (Figure 2): Group I (*RsIPT1*, *8-1*, *8-2*), Group II (*RsIPT2*), Group III (*RsIPT3-1*, *3-2*, *5-1*, *5-2*, *7-1*, *7-2*), and Group IV (*RsIPT9-1*, *9-2*). This classification of *RsIPT* genes was consistent with their homologues in other genetically close species. Most *IPT* genes had one corresponding gene in *Arabidopsis*, two in *R. sativus*, *B. rapa*, *B. oleracea*, and *B. nigra*, and four in *B. napus*, while *RsIPT2* had two in *B. napus* and only one copy in *Arabidopsis* and other diploid species. There were no corresponding genes for *AtIPT4* and *AtIPT6* in radish or other *Brassica* species, suggesting that these genes might have been lost in the divergence process from *Arabidopsis*. *RsIPT10* was not clustered into any group, suggesting that this gene may be unique to radish. The *RsCKX* genes were classified into four subgroups (Figure 3): Group I (*RsCKX1-1*, *1-2*, *1-3*, *6*), Group II (*RsCKX2-1*, *2-2*, *3-1*, *3-2*, *4*), Group III (*RsCKX5*), and Group IV (*RsCKX7-1*, *7-2*). Similar to *IPT* genes, most *CKX* genes also had one corresponding gene in *Arabidopsis*, two in radish and other diploid species, and four in *B. napus*. Moreover, the homologues of *AtCKX1* contained three paralogous genes in diploid species and six in *B. napus*. Although there were various degrees of gene loss and gene duplication in the process of speciation in *Arabidopsis*, the number of *IPT* and *CKX* genes in *Raphanus* and *Brassica* species was approximately twice as high as in *Arabidopsis*.

### 2.3. Gene Structure and Conserved Motif Analysis of the RsIPT and RsCKX Genes

**Figure 4:**
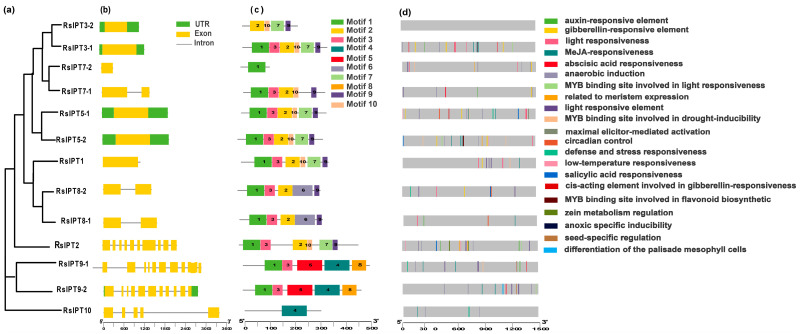
Phylogenetic relationship (**a**), gene structure (**b**), conserved motifs (**c**), and cis-acting elements in promoters (**d**) of *IPT* genes in radish. The yellow rectangles and black lines represent exons and introns of genes, respectively, and their lengths are presented proportionally. The 10 conserved motifs and 21 cis-acting elements are indicated in different colors.

**Figure 5:**
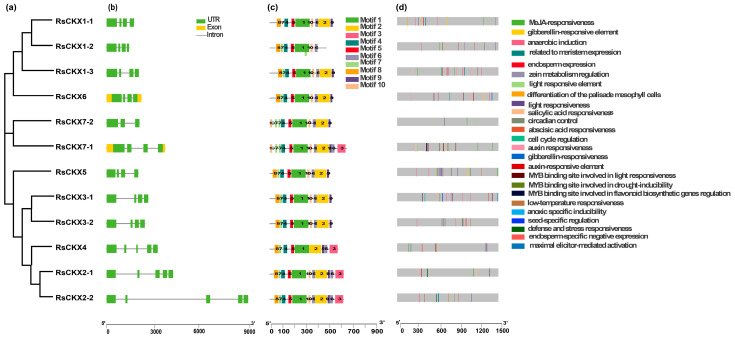
Phylogenetic relationship (**a**), gene structure (**b**), conserved motifs (**c**), and cis-acting elements in promoters (**d**) of *CKX* genes in radish. The green rectangles and black lines represent exons and introns of genes, respectively, and their lengths are presented proportionally. The 10 conserved motifs and 25 cis-acting elements are indicated in different colors.

To further understand the *IPT* and *CKX* genes, the exon-intron structure of these genes was analyzed. Most *RsIPT* genes (66.67%) had one or no introns (*RsIPT1*, *3*, *5*, *7*, *8*), while another one-third had more than nine introns (*RsIPT2* and *RsIPT9*) (Figure 4b). Compared to the *IPT* family, the *CKX* family presented more diversity (Figure 5b,c). Not only did most *RsCKX* genes have three or four introns, but the length of the intron also varied among groups. However, the genes that were classified into the same group had similar structures, especially the number and length of exons that were relatively conserved.

Then, to further reveal protein functional diversification, 10 conserved motifs in the *RsIPT* and *RsCKX* genes were predicted using MEME software. The results showed similar motif compositions in the same groups (Figure 4d and Figure 5d). For instance, group I (*RsIPT*) contained motifs 1, 2, 3, 6, and 9; group III (*RsIPT*) contained motifs 1, 3, 2, 10, 7, and 9; and group II (*RsCKX*) contained motifs 1, 2, 3, 4, 5, 6, 7, 8, 9, and 10. However, some genes lacked motifs in the identical group. In group III (*RsIPT*), *RsIPT3-2* lacked motifs 1 and 3, *RsIPT7-1* lacked motif 7, and *RsIPT7-2* only had motif 1. In group I (*RsCKX*), *RsCKX1-2* lacked motifs 2 and 9. In group II (*RsCKX*), *RsCKX3-1/3-2* lacked motif 3. Taken together, the motif composition in all *RsCKX* genes was very similar, except in *RsCKX 7-1/7-2*, which lacked motif 8 (common motifs 1, 4, 5, 6, 7, 9, and 10), while the common motifs were only motifs 1 and 3 in most *RsIPT* genes. The *RsCKX* genes exhibited a similar motif composition, suggesting that these proteins might have similar functions. In general, the variation trend of the exon–intron structure and motifs of the *RsIPT* and *RsCKX* genes aligned with their phylogenetic classification.

### 2.4. Cis-Acting Elements in the Promoters of RsIPT and RsCKX Genes

To obtain the potential functions and transcriptional regulation of *RsIPT* and *RsCKX* genes, the cis-regulatory elements in the promoters were identified. In total, 21 cis-regulatory elements were obtained in the *RsIPT* genes (Figure 4d). Cis-regulatory elements related to light responsiveness, such as GT1-motif, AAAC-motif, Sp1, G-box, and MRE, were most widely distributed. Among phytohormone-related elements, auxin-responsive elements (TGAs) were identified in five *RsIPT* genes; salicylic acid (SA) responsiveness (TCA) was identified in four *RsIPT* genes. Abscisic acid responsiveness (ABRE) and gibberellin-responsive elements, such as the P-box and GARE motif, were identified in seven *RsIPT* genes. Methyl jasmonate (MeJA) responsiveness (TGACG-motif and CGTCA-motif) was identified in eight *RsIPT* genes. At least one of the phytohormone-related elements was present in each *RsIPT* gene. This result shows that the *RsIPT* genes are extensively associated with phytohormone metabolism and signal transduction, playing an important role in plant growth and development. Stress-related elements, such as anaerobic induction (ARE), low-temperature responsiveness (LTR), MYB binding site involved in drought inducibility (MBS), MYB binding site involved in flavonoid biosynthetic (MBSI), and defense and stress responsiveness (TC-rich repeats), were also widely distributed among *RsIPT* genes. This suggests that *RsIPT* genes are responsive to multiple stimuli and play a part in defense to abiotic and biotic stress. In addition, identified elements related to differentiation of the palisade mesophyll cells (HD-Zip 1), seed-specific regulation (RY-element), meristem expression (CAT-box), and zein metabolism regulation (O2-site) further illustrated the functional diversification.

Compared to the *RsIPT* genes, 25 cis-regulatory elements were identified in the *RsCKX* genes (Figure 5d). Auxin responsiveness (AuxRR-core), cell cycle regulation (MSA-like), endosperm expression (GCN4_motif), and endosperm-specific negative expression (AACA_motif) were only predicted in the *RsCKX* genes. All genes contained light-responsive elements, but the types of elements differed from the *RsIPT* genes. Specifically, the 3-AF1 binding site and ACE elements were unique to the *RsCKX* genes, and the AAAC motif was only identified in the *RsIPT* genes. The hormone-related cis-elements were widely distributed, indicating that the *RsCKX* genes also play a vital role in plant growth and development processes in which hormones participate. Similarly, the *RsCKX* genes play an important part in the stress response due to their stress-related elements.

### 2.5. Gene Duplication and Divergence of the RsIPT and RsCKX Genes

**Figure 6:**
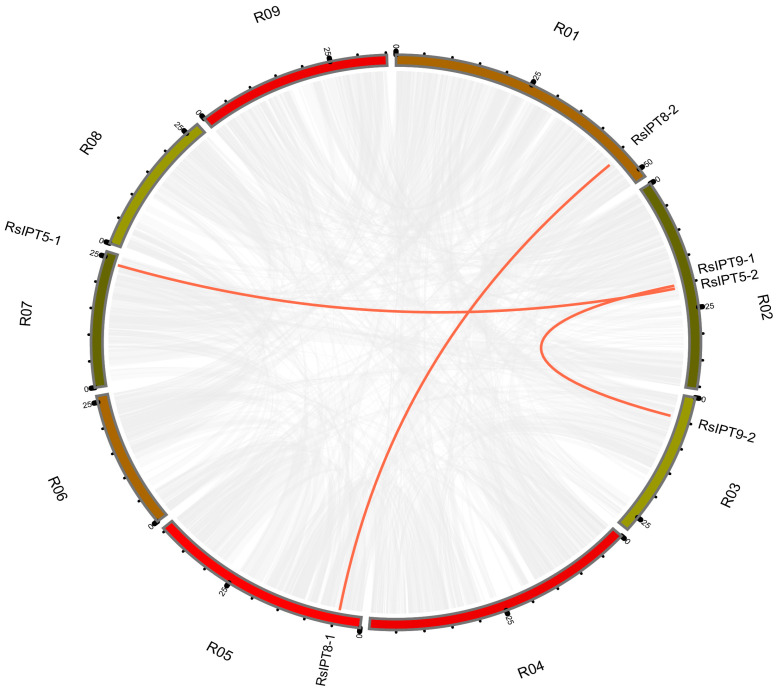
Collinearity analysis of *RsIPT* genes among radish chromosomes. Different chromosomes are indicated with corresponding number and colors in different regions of circle. The genes connected by the red line are segmental duplication pairs.

**Figure 7:**
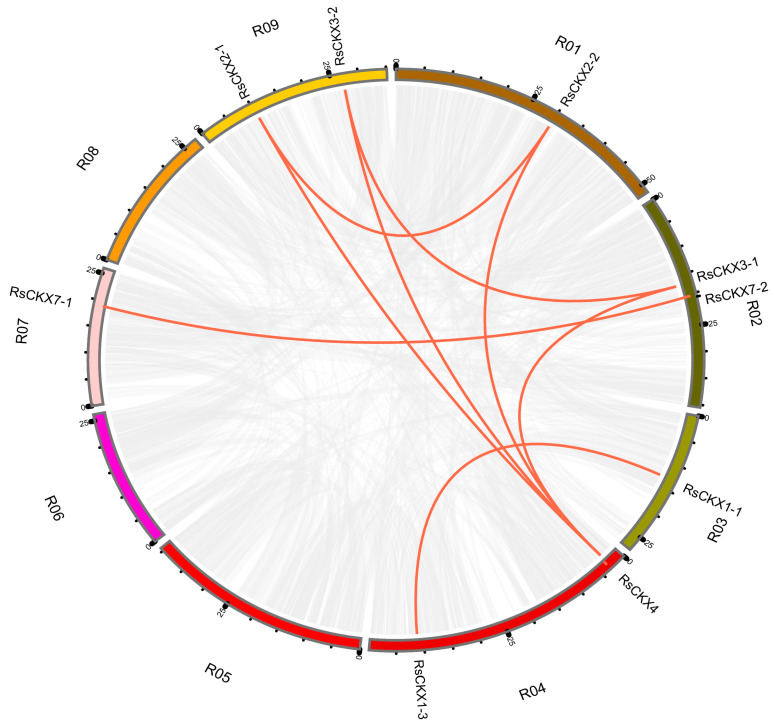
Collinearity analysis of *RsCKX* genes among radish chromosomes. Different chromosomes are indicated with corresponding number and colors in different regions of circle. The genes connected by the red line are segmental duplication pairs.

To comprehend the expansion of the *RsIPT* and *RsCKX* genes and evolutionary mechanisms, gene duplication events were analyzed. Generally, tandem and segmental duplications are the major patterns of gene duplication. In this study, MCScanX and BLAST were used to search for tandem and segmental duplications. The results are shown in Figure 6 and Figure 7. Although the result revealed no tandem duplicated genes, segmental duplication existed in the two gene families. In the *RsIPT* genes, three segmental duplicate pairs were obtained: *RsIPT5-1/RsIPT5-2*, *RsIPT8-1/RsIPT8-2*, and *RsIPT9-1/RsIPT9-2*. Moreover, there were eight gene pairs with segmental duplication in *RsCKX* genes: *RsCKX1-1/RsCKX1-3*, *RsCKX2-1/RsCKX2-2*, *RsCKX2-1/RsCKX4*, *RsCKX3-1/RsCKX3-2*, *RsCKX3-1/RsCKX4*, *RsCKX3-2/RsCKX4*, *RsCKX4/RsCKX8*, and *RsCKX7-1/RsCKX7-2*. Six genes also presented trinal pairs: *RsCKX3-1*, *RsCKX3-2*, and *RsCKX4*; and *RsCKX2-1*, *RsCKX2-2*, and *RsCKX4*. Based on these results, segmental duplication was the major type of gene duplication causing expansion and played a key role in *RsIPT* and *RsCKX* gene evolution.

To understand the divergence of *IPT* and *CKX* genes in *Raphanus*, the Ka and Ks values of orthologous and paralogous genes of both gene families were obtained (Table 3 and Table S1). The Ks values of orthologous genes between *A. thaliana* and *R. sativus* ranged from 0.32 to 0.62, suggesting that the divergence time of the *RsIPT* and *RsCKX* genes from *A. thaliana* was 10–21 MYA (Table 3). Comparative analysis indicated that the divergence times of two orthologous copies of the *IPT* and *CKX* genes in *A. thaliana* were nearly consistent. The Ks values of orthologous genes between *B. rapa* and *R. sativus* ranged from 0.08 to 0.51 (Table S2a, but the Ks value among orthologous copies of the same *RsIPT* or *RsCKX* genes varied distinctly. Therefore, the divergence times were classified into two periods: 2–10 and 10–17 MYA (Table S2b). Compared with the corresponding orthologous genes, the Ks values of paralogous genes in radish were lower, suggesting that the duplication of *IPT* and *CKX* genes in radish was due to divergence from *A. thaliana*. In addition, the Ka/Ks of orthologous and paralogous genes were significantly smaller than 1, suggesting that the genes of these two families experienced purifying selection during evolution.

### 2.6. Expression Profiles of RsIPT and RsCKX Genes in Tissues and Growth Stages of Radish Root

**Figure 8:**
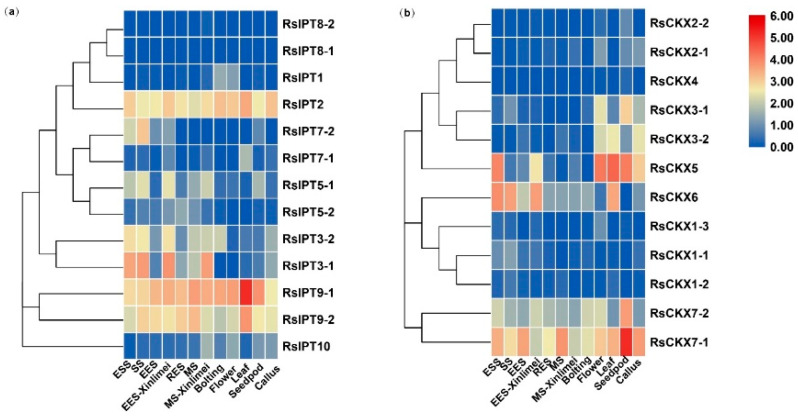
Expression profiles of (**a**) *RsIPT* and (**b**) *RsCKX* genes in different tissues and root growth stages in XYB36-2 and Xinlimei radish. ESS: seedling stage; SS: splitting stage; EES: early expanding stage; RES: rapid expanding stage; MS: mature stage. Color scale indicates the relative expression of genes by log_2_ transformation compared with the controls.

To determine the expression of *RsIPT* and *RsCKX* genes among different tissues and root development stages, the early transcriptional data of tissues, including roots, stems (the bolting stems), leaves, flowers, siliques, and callus and seedling stage (ESS), splitting stage (SS), early expanding stage (EES), rapid expanding stage (RES), and mature stage (MS) of roots, were used to detect the expression profiles of *RsIPT* and *RsCKX* genes. The *RsIPT* and *RsCKX* genes presented diversified expression profiles (Figure 8). Many genes in the same group presented similar expression patterns. Groups II (*RsIPT2*) and IV (*RsIPT9-1*, *RsIPT9-2*) were highly expressed in all tissues and root development stages, while group I (*RsIPT1*, *RsIPT8-1*, and *RsIPT8-2*) was almost not expressed in any tissues or root stages of root except *RsIPT1*, which had only low expression levels in the bolting stems and flowers. However, the group III genes showed differentiated modes of expression. *RsIPT3-1* and *RsIPT3-2* were highly expressed in roots but possessed low expression levels in calli. *RsIPT3-2* was also significantly expressed in the bolting stems. *RsIPT5-1* had a high expression level in roots, seedpods, and stems, while *RsIPT5-2* was only expressed in roots. *RsIPT7-1* and *RsIPT7-2* also exhibited organ-specific expression in leaves and roots, respectively. In addition, *RsIPT10* was mainly expressed in roots, flowers, seedpods, and calli. Compared with XB-36-2 (white freshy root), *RsIPT3-1*, *RsIPT3-2*, and *RsIPT5-1* were only expressed in EES-Xinlimei (red freshy root). These genes may play an important part in the early expansion of freshy roots in Xinlimei. As shown in Figure 8b, the number of expressed *RsCKX* genes was lower than that of *RsIPT* genes. The expression of genes that belonged to the same groups presented diversity. *RsCKX1-1* was expressed only in the roots, specifically in the root splitting stage. *RsCKX6* was expressed in leaves and roots and was mainly expressed in the early growth phases before expanding in the roots. However, *RsCKX1-2* and *RsCKX1-3* of the same group were not expressed in any tissues or root growth stages, and neither were *RsCKX2-1*, *RsCKX2-2*, or *RsCKX4* of group II. Another two genes (*RsCKX3-1* and *RsCKX3-2*) of group II were expressed in three tissue types (flowers, calli, seedpods, or leaves). Group III only contained one gene, *RsCKX5*, that was expressed in all tissues except stems. Similarly, *RsCKX7-2* was also expressed in all tissues except leaves. Only *RsCKX7-1* (group IV) was ubiquitously expressed in all tissues and root growth phases.

### 2.7. Expression Divergences of RsIPT and RsCKX Genes after Infection with P. brassicae

**Figure 9:**
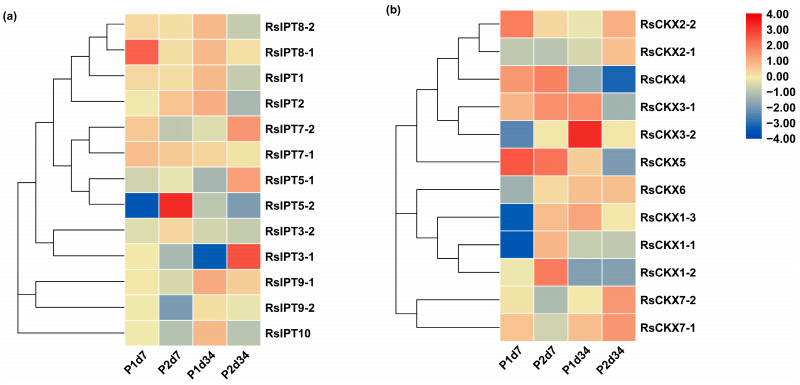
Expression profiles of (**a**) *RsIPT* and (**b**) *RsCKX* genes during different infection periods after inoculation with *Plasmodiophora brassicae* in susceptible (P1, YS-472) and resistant varieties (P2, YR-456). P1d7: susceptible variety 7 days after inoculation/corresponding control with no infection; P2d7: resistant variety 7 days after inoculation/corresponding control with no infection; P1d34: susceptible variety 34 days after inoculation/corresponding control with no infection; P2d34: resistant variety 34 days after inoculation/corresponding control with no infection. Color scale indicates the relative expression of genes by log_2_ transformation compared with the controls.

To investigate the response of *RsIPT* and *RsCKX* genes in *P. brassicae*-radish interactions in two infection periods, transcriptional data from eight treatments with primary infection (7 days after inoculation (dai)) and secondary infection (34 dai) of two different resistance materials were used to detect the expression patterns of *RsIPT* and *RsCKX* genes (Figure 9). *RsIPT1* and *RsIPT8-2* of group I were not expressed in roots; that is, there was no reaction after stimulation with *P. brassicae*. However, *RsIPT8-1*, another gene of group I, was upregulated in P1d7 (susceptible materials) compared with the corresponding control. *RsIPT2*, the only group II gene, had a fairly low expression level in both P1 and P2 at 7 and 34 dai. Similarly, most genes in Group III were also indistinctly expressed during primary infection (7 dai). However, during secondary infection, most of these genes were significantly downregulated in P1d34 but upregulated in P2d34. In group IV, although two genes (*RsIPT9-1* and *RsIPT9-2*) had a relatively low expression level in most treatments of the two periods compared with their corresponding control, *RsIPT9-1* was upregulated in P2d34, while *RsIPT9-2* was downregulated in P2d7. *RsIPT9-2* was the only gene that showed unapparent expression in P1d7 but significant downregulation in P2d7.

As shown in Figure 9b, the expression of *RsCKX* genes in group I presented a contrasting tendency at 7 dai (primary infection), while a consistent tendency was observed at 34 dai (secondary infection). Specifically, the genes were upregulated in P2d7 but downregulated in P1d7 after infection with *P. brassicae*. The genes of group I showed differential expression between susceptible and resistant materials during primary infection. In particular, *RsCKX1-2* was downregulated in susceptible P1d7 but was significantly upregulated in resistant P2d7. Two other genes (*RsCKX1-1* and *RsCKX1-3*) showed significant downregulation in susceptible P1d7 but near-significant upregulation in resistant P2d7. These genes may play a part in clubroot resistance in resistant materials by regulating CK degradation. The genes of group II had a similar expression tendency during primary infection and an inverse tendency during secondary infection. Although two genes (*RsCKX3-1* and *RsCKX4*) were significantly upregulated in P2d7, they were also similarly upregulated in P1d7 with no obvious distinction. However, *RsCKX2-2* showed differential expression between P1d34 and P2d34 and revealed a near-significant upregulation in P2d34 and a slight downregulation in P1d34. Similarly, *RsCKX7-1* and *RsCKX7-2* were also upregulated in P2d34. These genes may take part in the clubroot resistance of P2 and play a more important role in secondary infection. Overall, the *RsCKX* genes were more expressed than the *RsIPT* genes and may play a more important role in resistance to *P. brassicae*.

**Figure 10:**
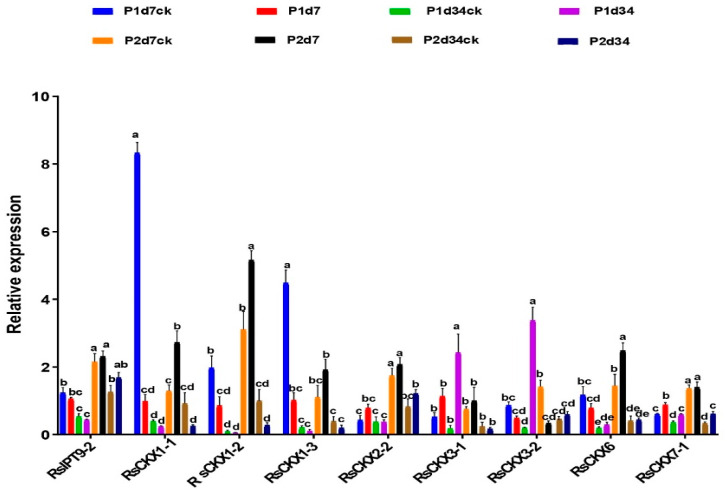
Expression levels of selected *RsIPT* and *RsCKX* genes in eight treatments of susceptible and resistant materials under *Plasmodiophora brassicae* stress by qRT-PCR analysis. P1d7: susceptible variety 7 days after inoculation; P1d7CK: susceptible variety 7 days with no infection; P2d7: resistant variety 7 days after inoculation; P2d7CK: resistant variety after 7 days with no infection; P1d34: susceptible variety 34 days after inoculation; P1d34CK: susceptible variety after 34 days with no infection; P2d34: resistant variety 34 days after inoculation; P2d34CK: resistant variety after 34 days with no infection. Different lowercase letters indicate significant differences (*p* < 0.05).

To validate the expression of *RsIPT* and *RsCKX* genes after infection with *P. brassicae*, genes that might be related to clubroot resistance were selected to investigate their expression level based on qRT-PCR. As shown in Figure 10, the expression levels of these genes were consistent with previous transcriptional data. Four genes (*RsCKX1-1*, *RsCKX1-2*, *RsCKX1-3*, and *RsCKX6*), which all belonged to group I, showed a higher expression level in the resistant variety and a lower expression level in the susceptible variety during the primary infection period.

### 2.8. Predicted Secondary Structure and 3D Structure of RsCKX Proteins

The *RsCKX* genes might be more involved in clubroot resistance than the *RsIPT* genes. Thus, the high-level protein structures of these genes were further predicted due to their similar biological functions. According to the SOMPA secondary structure prediction method, nine states of secondary structures were forecasted (Table 4). The secondary structure of all *RsCKX* proteins mainly included an alpha helix (Hh), an extended strand (Ee), a beta turn (Tt), and a random coil (Cc). The random coil had the highest proportion among the four types, followed by the α-helix and the extended strand. In comparison, the β-turn was the least. From the perspective of the subfamily, there were similar types of secondary structures, and no significant differences in the proportion of different states were observed among the four subfamilies.

**Figure 11:**
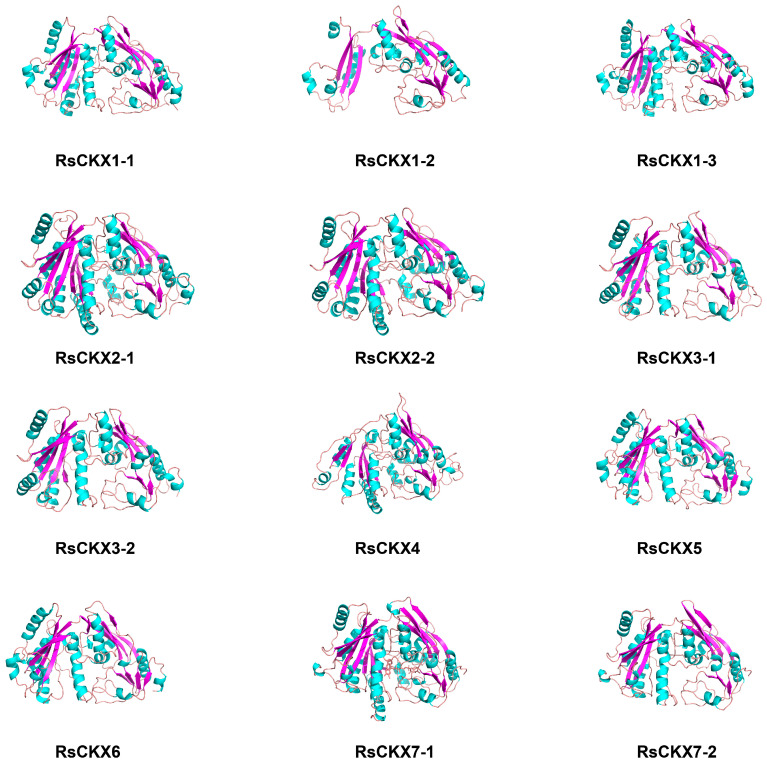
3D structure of 12 *RsCKX* proteins. The secondary structure in green represents an alpha helix, purple represents an extended strand, and pink represents a random coil.

The 3D structure of these proteins was further developed. In terms of the result of PSI-BLAST, the sequence identity between *RsCKX* proteins and their target templates, which were searched from the PDB database, was greater than 30%. Therefore, the 3D structure of *RsCKX* proteins was predicted based on the homology modeling method. As shown in Figure 11 by the 3D structure of *RsCKX* proteins, the secondary structure contents of the modeled 3D structure were generally consistent with those above, consisting mainly of random coil, alpha helix, and extended strand. Further analysis revealed that the RMSD value of alignment between different 3D structures of *RsCKX* proteins was less than 1 Å, suggesting that all *RsCKX* proteins had similar 3D structures (Table 5). Moreover, the RMSD values between the proteins belonging to the same group were closer to 0 than the proteins belonging to different groups. Thus, the 3D structure of the *RsCKX* proteins that were classified into the same group was more similar.

### 2.9. Analysis of the Interaction Networks of RsCKX Proteins

**Figure 12:**
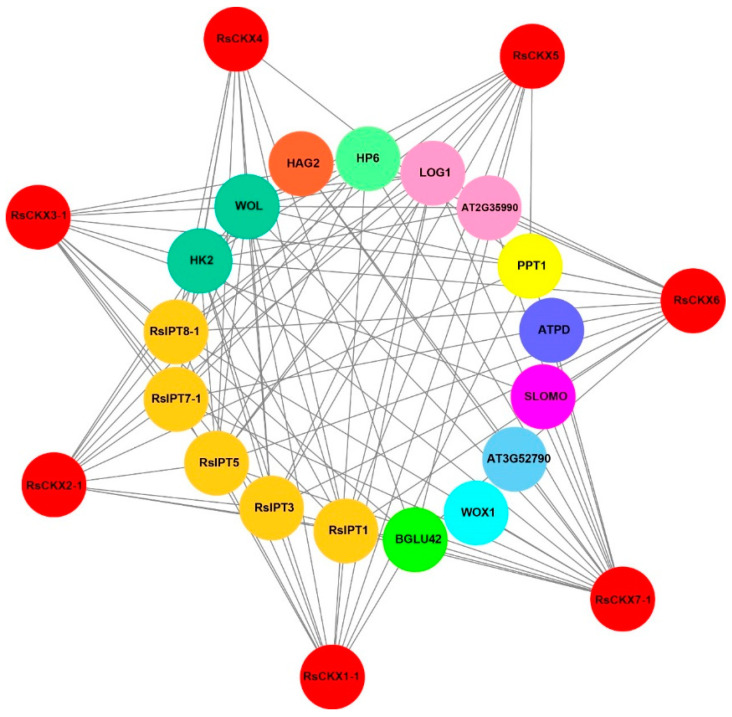
Protein-protein interaction networks of *RsCKX* proteins with other proteins predicted using STRING. The red circles represent *RsCKX*s, and the inner circles represent proteins interacting with *RsCKX*s. The different colors indicate that the proteins belong to different families. Different colors including brown, lime, aqua, turquoise, purple, blue, yellow, pink, spring green, orange, and light green represent cytokinin synthase, beta-glucosidase, wuschel related homeobox 1, peptidoglycan-binding LysM domain-containing protein, F-box/LRR-repeat protein 15, ATP synthase, Polyprenyltransferase 1, cytokinin riboside 5′-monophosphate phosphoribohydrolase, histidine-containing phosphotransfer protein, histone acetyltransferase, and cytokinin receptors. The gray lines indicate interactions.

To better determine the regulatory network of CKX proteins and their biological function, their protein-protein interaction relationships were predicted using an orthology-based method [43,44]. Seven *RsCKX* genes have orthologous relationships with *Arabidopsis*. Furthermore, 20 interactors were found for these CKX proteins in *Arabidopsis*. Unlike *Arabidopsis*, only 17 interactors were identified for corresponding CKX proteins based on orthologous relationships (Figure 12). There were no interactions between the IPT4, IPT6, and HK3 proteins and CKX proteins in radish.

Specifically, interactors with CKX proteins were divided into 11 categories in radish (Figure 12). There were five interacting proteins belonging to the IPT family among the CKX interactors. Other families had only one or two members. The functions of proteins interacting with CKX proteins were further analyzed. These proteins had abundant functions involved in CK biosynthesis (RsIPTs, LOG1, AT2G35990), initiating vascular primordium (WOX1), cell wall macromolecule catabolic process (AT3G52790), coenzyme Q (CoQ) biosynthesis (PPT1), inhibitor of CK signaling (HP6), auxin homeostasis (SLOMO), and transmission of the stress signal (HK2, WOL). Specifically, CKXs interacted with the BGLU42 protein, which was involved in the secretion of root-derived phenolics upon iron ion (Fe) depletion and promoted disease resistance.

## 3. Discussion

An increasing number of studies have found that CKs play a very important role in plant growth, development, and resistance [12,38,45]. IPTs and CKXs are key enzymes catalyzing CK synthesis and degradation, respectively. In this study, 13 *IPT* and 12 *CKX* genes were identified in *R. sativus* (2n = 18). Moreover, the number of genes was similar in diploid *Brassica* species and double in allotetraploid *B. napus* (2n = 38). These identified *IPT* and *CKX* genes were essentially consistent with previous studies in *Arabidopsis* (2n = 10) and *B. rapa* (2n = 20) [32,36,40,41].

Previous studies have revealed that angiosperms, including *Brassicaceae* species, have undergone multiple rounds of whole-genome duplication (WGD) [46,47,48,49,50,51,52]. *Raphanus* and *Brassica* species have both undergone whole-genome triplication (WGT) [50,52,53]. Moreover, the triplication processes were followed by diploidization, substantial genome rearrangements, and differential gene losses [54]. In our study, most *IPT* and *CKX* genes in radish were retained, maintaining around twice the number in *Arabidopsis*, suggesting that *IPT* and *CKX* genes also experienced a diploidization process following WGT events. Typically, the increase in genes was mainly by means of gene duplication, which occurred as tandem and segmental duplication. For the *RsIPT* and *RsCKX* genes identified in this study, gene expansion occurred through segmental duplication, with no tandem-duplicated genes. Similar results have been reported in *B. rapa* [36]. The varying degrees of gene losses are usually followed by polyploidization, except in the IPT and CKX families. For example, although most genes retained two copies, some genes, such as *RsIPT2* and *RsCKX5*, only retained one copy. Two IPT genes (*AtIPT4* and *AtIPT6*) were entirely lost and not identified in *Raphanus* and other *Brassica* species.

The synonymous substitution rate (Ks) between homologous genes was thought to be a measure to explore the timing of duplication events and the divergence of genes [55,56]. Based on the Ks values of orthologous genes, the comparative analysis of *Arabidopsis* with *R. sativus* or *B. rapa* indicated that the split from their common ancestor, *Arabidopsis*, occurred 13–29 MYA, and they shared a recent WGT event estimated at 10–21 MYA [52,54]. We investigated the divergence for orthologous genes of the IPT and CKX families between *Arabidopsis* and *Raphanus* and found that the divergence of these genes from their orthologs in *Arabidopsis* occurred 10–21 MYA, suggesting that the divergence of *IPT* and *CKX* genes occurred after the speciation of *Raphanus* from *Arabidopsis* and during the WGT event. In addition, the divergence of paralogous genes in radish occurred after the divergence of orthologous genes. The divergence time of orthologous genes between *B. rapa* and *R. sativus* was obviously later than the divergence time from *Arabidopsis* and nearly consistent with the split of *Raphanus* from *Brassica* species, which occurred during the WGT event, according to previous studies [52,57]. Therefore, the divergence of *IPT* and *CKX* genes between *Raphanus* and *Brassica* may be consistent with the divergence of the two species.

The exon–intron gene structure was an essential element that played an important role in evolution and was related to gene function and expression level [58,59,60]. In terms of the number of introns or exons, the *RsIPT* genes were classified into two gene categories. One group had one or no introns (*RsIPT1*, *3*, *5*, *7*, *8*), and the other category had more than nine introns (*RsIPT2* and *RsIPT*9). Similar exon–intron structures of *IPT* genes were identified in Chinese cabbage, apples, and nine *Rosaceae* species [36,61,62]. The variation in gene structure resulted in a diversity of functions. According to their orthologous genes in *Arabidopsis* and *B. rapa*, two categories of exon–intron structures corresponded to different types of *IPT* genes: ATP/ADP IPT (*RsIPT1*, *3*, *5*, *7*, *8*) and tRNA *IPT* genes (*RsIPT2* and *RsIPT*9) [32,33]. We found that the tRNA *IPT* genes, which were rich in introns, presented a higher level of expression in tissues than the ATP/ADP *IPT* genes. The highly expressed genes had more introns and were also present in rice and *Arabidopsis* [60,63]. In contrast, the gene structures of the *RsCKX* genes were relatively similar in the number of introns, whereas they also had fewer introns. The genes with few introns could rapidly respond to the challenges of stress in early studies [38,64,65]. Some stress-related gene families had a lower number of introns, such as the LRR family, Hsp20 family, and chitinase family [65,66,67]. In this study, most *RsIPT* and *RsCKX* genes contained few introns, suggesting that these genes might be kept ahead in transcription and activation of stress responses. In addition, the corresponding types of cis-elements in the promoters helped regulate transcription and expression to cope with stress. Many cis-elements related to phytohormones, defense, and stress, such as LTR (CCGAAA), ABRE (ACGTG), TGA (AACGAC), TCA (TCAGAAGAGG), CGTCA-motif, and TGACG-motif, were searched in the promoters of the *RsIPT* and *RsCKX* genes. These cis-elements have also been found to take part in resistance to biotic and abiotic stress [68,69,70]. The TGACG-motif binding factor (TGA) is a conserved transcription factor involved in the regulation of MeJA and SA signaling pathways as well as in the disease resistance response [71,72]. In *Arabidopsis*, CK can augment resistance to *Pst* (*Pseudomonas syringae pv. tomato DC3000*) by modulating the binding of ARR2/*TGA3* with the *PR-1* promoter [73]. A similar study has shown that *CmTGA3*, *CmTGA8*, and *CmTGA9* also enhanced the resistance to *Pst* in melon [74]. Other studies have also revealed that the *TGA* gene is related to M7SB41-mediated host plant powdery mildew resistance [75]. The transcription factor *PvNAC52* has been reported to specifically bind to ABRE and enhance transgenic *Arabidopsis* resistance to abiotic stress, such as salt and alkali stress [76]. The arrangement that responded to stress in the organization of these genes might also be conducive to *P. brassicae*.

Both the *RsIPT* and *RsCKX* genes had a wide variety of expression patterns among tissues. For example, *RsIPT3-1*, *3-2*, *5-2*, and *RsCKX1-1*, *6* were primarily expressed in nutritive organs, while *RsIPT1* and *RsCKX2-1*, *2-2*, *3-1*, *3-2*, *5* were primarily expressed in reproductive organs, which were similar to their orthologous genes in *B. rapa* [36]. Similarly, the genes *RsIPT2* and *RsIPT9-1/9-2*, as well as *AtIPT2*, *AtIPT9*, *BrIPT2*, and *BrIPT9-1*/*9-2*, were ubiquitously expressed [33,36]. However, the *BrCKX4* gene was highly expressed in the vegetative organs of Chinese cabbage, while the expression of *RsCKX4* was barely detected in either the vegetative or reproductive organs. Expression differentiation was also presented in duplicate gene pairs. In Chinese cabbage, *BrIPT5-1/5-2* and *BrIPT7-1/7-2* were expressed in the same tissues, while the corresponding orthologous gene pairs were expressed in different tissues in radish. The expression of gene pair *CKX1-1/1-2/1-3* also differed in Chinese cabbage and *radish*. The expression discrepancy of segmentally duplicated gene pairs of the *IPT* and *CKX* genes was also identified in other species [61,77]. Compared to *B. rapa*, the expression patterns of the corresponding genes in *R. sativus* were more abundant, suggesting that they might have more diversified functions.

Transcriptome analysis has shown the key role of CK in response to *P. brassicae* in *Brassicaceae* [21,38]. The levels of CK are mainly regulated by the IPT and CKX families. According to RNA-Seq in this study, although the *RsIPT*9 gene was downregulated in resistant materials, it was not significantly upregulated in susceptible materials. Further verification using qRT-PCR also showed that this gene had no significant expression differences between the two types of materials. This was also true for other *RsIPT* genes. Compared with the *RsIPT* genes, the *RsCKX* genes of group I presented differential expression between resistant and susceptible materials during primary infection. The *RsCKX* genes, but not the *RsIPT* genes, played a dominant role in regulating the CK level in response to pathogen infection. In particular, the *RsCKX1-1*/1-2/1-3 genes were upregulated in resistant materials but downregulated in susceptible materials during primary infection. Moreover, overexpression lines of the orthologous gene *AtCKX1* have been shown to significantly alleviate clubroot symptoms and disease resistance in *Arabidopsis thaliana* [21]. Therefore, the *RsCKX1-1*/1-2/1-3 genes may play an important role in clubroot resistance in radish. Further promoter analysis showed that they all included two MeJA-responsiveness cis-acting elements (CGTCA/TGACG-motif), suggesting that they may participate in the interaction of CK signaling with the JA pathway. In addition, the MeJA-responsiveness elements were also identified in genes of the *CKX* family in *B. oleracea* [38]. Previous studies have indicated that CK takes part in SA-mediated resistance to the fungus *Alternaria brassica* in *Arabidopsis* [72]. Therefore, we assumed that the CK–JA interaction may have an important impact on clubroot resistance.

Previous studies have shown the 3D structure of TaPYL proteins in wheat is similar and rather conserved [44]. In our study, the 3D structures of RsCKX proteins were also similar (RMSD < 1 Å). The 3D structures of RsCKX proteins in the same group were closer (RMSD < 0.2 Å), especially the 3D structure of proteins of *RsCKX1-1*/1-2/1-3 (RMSD < 0.1 Å). Further, the 3D structures of RsCKX proteins were basically consistent with the results of phylogenetic, gene structure, and conserved motifs analysis. This indicated that the 3D structures of RsCKX proteins were also relatively conservative. The proteins that interacted with RsCKX proteins possessed multiple biological functions involving CK biosynthesis (RsIPTs, LOG1, AT2G35990), inhibitor of CK signaling (HP6), auxin homeostasis, transmission of the stress signal (HK2, WOL), and disease resistance (BGLU42). Previous studies have shown that the alteration of CKs and auxin homeostasis mediates clubroot formation [20,21,22,23,24,25,26]. The RsCKX proteins may play an important role by interacting with the proteins involved in the CK pathway and auxin homeostasis. In addition, the overexpression of β-glucosidase BGLU42, which was a MYB72-dependent key regulator, led to constitutive systemic resistance in *Arabidopsis* roots [78]. The RsCKX proteins interacted with BGLU42, which may be beneficial for participating in clubroot resistance.

## 4. Materials and Methods

### 4.1. Identifying IPT Genes and CKX Genes

The sequences of *R. sativus*, *B. rapa* (version 3.0), *B. oleracea* (JZS version 2.0), *B. napus* (ZS11H), and *B. nigra* (version 2.0) were downloaded from the Brassicaceae Database (BRAD, http://brassicadb.cn, accessed on 25 October 2023) [79]. IPT and CKX protein sequences of *Arabidopsis*, which were downloaded from NCBI (https://www.ncbi.nlm.nih.gov/, accessed on 25 October 2023), were used as a query to search *IPT* and *CKX* gene families in the studied species using BLASTP (1 × 10^−10^). In addition, the hmmsearch program (HMMER 3.0) was utilized to screen protein sequences according to the hidden Markov model (HMM) of the CK-bind domain (Pfam: PF09265) and isopentenyl transferase domain (PF01745) with an E-value of 1 × 10^−5^ [80]. The results were integrated, and conserved domains were verified using CDD (https://www.ncbi.nlm.nih.gov/Structure/cdd/wrpsb.cgi, accessed on 26 October 2023) and Pfam (http://pfam.xfam.org/, accessed on 26 October 2023) [81,82]. For the *RsIPT* and *RsCKX* genes, subcellular localization predictions were performed using WoLF PSORT (https://wolfpsort.hgc.jp/, accessed on 26 October 2023) [83].

### 4.2. Mapping IPT and CKX Genes to Chromosomes

Chromosome maps were constructed using the location of all *CKX* and *IPT* genes based on the chromosome length with MapChart software (version 2.32, https://www.wur.nl/en/show/Mapchart.htm accessed on 19 November 2023) [84]. The location of all genes on the chromosomes was visualized according to their start position.

### 4.3. Phylogenetic Tree Construction of RsIPT and RsCKX Gene Families

Multiple sequence alignments of all amino acid sequences were performed using MUSCLE program of MEGA (version 7.0, https://www.megasoftware.net accessed on 19 November 2023) [85]. Phylogenetic analysis was conducted using the neighbor-joining (NJ) method of the MEGA program [86]. The NJ tree was constructed using the Jones–Taylor–Thornton (JTT) model with 500 bootstrap replicates. The online tool Evolview (https://evolgenius.info//evolview-v2/#login, accessed on 29 October 2023) was used for further color-coding of branches and protein names to clearly classify proteins [87].

### 4.4. Analysis of Gene Structure and Conserved Motif

The exon–intron structures of these *RsIPT* and *RsCKX* genes were obtained using the genome annotation files downloaded from BRAD and analyzed using the Gene Structure Display Server (GSDS, https://gsds.gao-lab.org/ accessed on 19 November 2023) [88]. The motifs of the *RsIPT* and *RsCKX* genes were obtained from the MEME program (http://meme-suite.org/index.html, accessed on 5 November 2023) [89].

### 4.5. Analysis of the Promoters of RsIPT and RsCKX Genes

The 1500 base pair (bp) DNA sequences of initiation codons upstream of the *RsIPT* and *RsCKX* genes were extracted and submitted to PlantCARE (http://bioinformatics.psb.ugent.be/webtools/plantcare/html, accessed on 6 November 2023) to identify cis-acting elements [90]. Then, the results were visualized together with the phylogenetic tree using TBtools (version 2.096) [91].

### 4.6. Analysis of Segmental Duplication, Tandem Duplication, and Collinearity between Raphanus and Arabidopsis or B. rapa

The tandem and segmental duplication events in radish were analyzed using the Multiple Collinearity Scan toolkit (MCScanX, https://github.com/wyp1125/MCScanX/, accessed on 10 November 2023) [92]. Gene duplication was confirmed using the BLAST program by following the criteria referred to by Zhao et al. [65]. The collinearity relationship between *Raphanus* and *Arabidopsis*, or *B. rapa*, was analyzed using MCscan (https://github.com/tanghaibao/jcvi/wiki/MCscan, accessed on 13 November 2023). The KaKs_Calculator was used to calculate the non-synonymous (Ka) and synonymous substitution rates (Ks) of duplicated *RsIPT* and *RsCKX* genes [93]. The divergence times of orthologous and paralogous genes were estimated using the formula T = Ks/2r, where r indicates the divergence rate of nuclear genes from plants. The r value is 1.5 × 10^−8^ synonymous substitutions per site per year for dicotyledonous plants [94].

### 4.7. Plant Materials, Cultivation, and Treatment with P. brassicae

Generally speaking, the fresh root of radish is composed of hypocotyl and root. The root was susceptible to *P. brassicae*. Two high-generation radish inbred lines, ‘YR-456’ (P2) and ‘YS-472’ (P1), were provided by the National Mid-term Genebank of Vegetable Germplasm Resources in Beijing (NMGVGR). ‘YR-456’ was highly resistant, while ‘YS-472’ was susceptible to *P. brassicae* race 4. Thirty germinated seeds were sown in plastic pots with a 10-cm diameter and filled with substrate, which consisted of a sterile peat–vermiculite mixture (1:1, *v*:*v*). All pots were placed in the identification chamber at 20–25 °C with a 14-h photoperiod. The treatment and control contained three biological replicates and 10 seedlings for each replicate (40 additional seedlings for each treatment were planted to observe the incidence of clubroot). Based on the previous research and the growth of seedlings, 20-day-old seedlings were inoculated with race 4 (2 × 10^8^ spores/mL, 5 mL), and inoculation was performed following the two-stage combined inoculation method [3]. The control was inoculated with an equal volume of sterile water. Subsequently, three seedlings were observed every three days from the first day after inoculation. Finally, the roots of ‘YR-456’ (P2) and ‘YS-472’ (P1) materials at 7 (primary infection) and 34 dai (secondary infection) (eight treatments including the corresponding control) were collected for RNA extraction, transcriptional analysis, and qPCR analysis.

### 4.8. RNA Extraction, cDNA Synthesis, qRT-PCR, and Statistical Analysis

The total RNA of 24 radish samples was isolated using a plant RNA Kit (QIAGEN, Dusseldorf, North Rhine-Westphalia, Germany) following the manufacturer’s instructions. Then, the cDNAs were synthesized by reverse-transcription with the Servicebio^®^RT First Strand cDNA Synthesis Kit (Servicebio, Wuhan, China). The specific primers for the *RsIPT* and *RsCKX* genes selected were designed using Primer 5 (Table S3). The *GAPDH* gene was employed as the reference gene. qPCR analysis was performed on the StepOnePlusTM Real-Time PCR System (Bio-RAD, Hercules, California, USA) and using 2×SYBR Green qPCR Master Mix (High ROX) (Servicebio, Wuhan, China) with a 15 µL reaction containing 7.5 µL of 2×SYBR Green qPCR Master Mix (High ROX), 0.75 µL of each primer (2.5 µM), 2.0 µL of cDNA, and 4 µL ddH_2_O. The cycling conditions were as follows: 95 °C for 10 min, followed by 40 cycles of 95 °C for 15 s, and 60 °C for 60 s. Analysis of the melting curve was carried out from 60 to 95 °C, with 0.3 °C every 15 s. The processing of expression levels was determined using the 2^−ΔΔCT^ method. Statistical analysis was performed in SAS (version 9.3) and used factorial analysis of variance (ANOVA) with Duncan’s multiple range test (PROC GLM statistical procedure) (*p* < 0.05).

### 4.9. Expression Profile Analysis of the RsIPT and RsCKX Genes

Early RNA-seq data from different tissues and root growth stages of root in radish (XB-36-2 and Xinlimei) were obtained from the NCBI database [95,96]. Therefore, the data for Xinlimei only contained the early, expanding, and mature root stages. The expression data from different infection periods of roots infected by *P. brassicae* were supplied by the State Key Laboratory of Vegetable Biobreeding (Table S4). The fragments per kilobase of transcript per million mapped reads (FPKM) values were used to reflect transcript abundance, and log2(FPKM) represented the gene expression values. Heat maps with the phylogenetic tree were merged using TBtools [91].

### 4.10. Prediction and Analysis of Secondary Structure and 3D Structure of Proteins

The high-level structures of proteins contributed to understanding their functions. Based on protein sequences, the online analysis tool SOPMA (https://npsa-pbil.ibcp.fr/cgi-bin/npsa_automat.pl?page=npsa_sopma.html, accessed on 18 November 2023) was applied to predict the secondary structure [97]. Then, homology modeling methods were used to predict the 3D structure of proteins. Specifically, the most similar homologies of *RsCKX* proteins were acquired using the positive-specific interactive basic local alignment search tools (PSI-BLAST) (http://www.ncbi.nlm.nih.gov/BLAST/, accessed on 19 November 2023) in the PDB database (https://www.rcsb.org/, accessed on 19 November 2023) [98,99]. The Swiss-model (https://swissmodel.expasy.org/ accessed on 19 November 2023) was utilized to search templates and build models for CKX proteins [100]. The predicted 3D structures were verified using the SAVES server (https://saves.mbi.ucla.edu/, accessed on 20 November 2023) [101,102,103,104]. In addition, the 3D structures of *RsCKX* proteins were aligned with each other using Superpose (http://superpose.wishartlab.com/, accessed on 22 November 2023) and PyMol (https://www.pymol.org/, accessed on 25 November 2023) [105]. The alignment results were measured by the root-mean-square deviation (RMSD) values. The closer the value was to 0, the more similar the structure was.

### 4.11. Interaction Network Analysis of RsCKX Proteins

To further explore the biological function and interaction relationship of the *RsCKX* proteins involved, Ortho Venn2 (https://orthovenn2.bioinfotoolkits.net/home, accessed on 28 November 2023) was employed to perform orthologous analysis with an E-value of 1 × 10^−2^ and an inflation value of 1.50 to identify the ortholog pairs between *RsCKX* and *AtCKX* [106]. The interaction network of *RsCKX* proteins was identified based on the orthologous genes between *Raphanus* and *Arabidopsis* using the STRING (https://string-db.org/, accessed on 29 November 2023) database with medium confidence (0.40) and no more than 20 interactors (the maximum number of interactors) [107].

## 5. Conclusions

This study comprehensively reported the gene structure, conserved domains, cis-elements in promoters, evolution patterns, 3D structure, and interaction network of the IPT and CKX families in the radish genome. Moreover, the expression profiles of the *RsIPT* and *RsCKX* genes in different tissues and root development stages, as well as their response to infection with *P. brassicae*, were also characterized. A total of 13 *IPT* and 12 *CKX* genes were identified and classified into four groups. The expansion mode of the *RsIPT* and *RsCKX* genes was segmental duplication. The divergence with their orthologs occurred after the speciation of *Raphanus* from *Arabidopsis* and during WGT. Purifying selection impelled the evolution of the genes of the two families. Further analysis showed that promoters of the *RsIPT* and *RsCKX* genes contained various cis-acting elements related to phytohormones and stress responsiveness. The expression profiles indicated that *RsCKX* genes played a more important role in the infection of *P. brassicae* than *RsIPT* genes, especially *RsCKX1-1*/1-2/1-3, which were significantly upregulated in resistant materials but downregulated in susceptible materials. The 3D structure of the *RsCKX* genes was predicted based on homology modeling. The protein interaction network further indicated that the *RsCKX* proteins played a key role in the response to stress and disease resistance.

## Figures and Tables

**Table 1:** Sequence characteristics and physicochemical properties of *RsIPT* genes in radish.

Gene Name	Gene ID	Chromosome	Gene Length (bp)	Protein Length (aa)	MW (Da)	PI	Predicted Localization
*RsIPT1*	*Rsa10009818*	R07	1032	344	38,962.4	9.75	Cytoskeleton
*RsIPT2*	*Rsa10020209*	R04	2169	470	53,202.4	6.18	Nucleus
*RsIPT3-1*	*Rsa10027751*	R05	1312	333	37,545	8.27	Chloroplast
*RsIPT3-2*	*Rsa10001423*	Scaffold638	1151	218	25,130.6	7.82	Cytoplasm
*RsIPT5-1*	*Rsa10015237*	R07	1926	335	37,836.2	5.78	Mitochondrion
*RsIPT5-2*	*Rsa10023282*	R02	1943	335	37,872	5.94	Chloroplast
*RsIPT7-1*	*Rsa10028124*	R01	1390	322	35,774.8	8.57	Chloroplast
*RsIPT7-2*	*Rsa10010488*	R05	339	113	12,356.3	10.35	Chloroplast
*RsIPT8-1*	*Rsa10042487*	R05	1563	327	36,928.9	9.29	Chloroplast
*RsIPT8-2*	*Rsa10027852*	R01	1405	325	36,904.9	9.08	Mitochondrion
*RsIPT9-1*	*Rsa10023211*	R02	3166	499	56,375.9	7.18	Chloroplast
*RsIPT9-2*	*Rsa10035858*	R03	2755	467	52,553.9	8	Chloroplast
*RsIPT10*	*Rsa10008422*	R02	3375	300	33,134	5.28	Chloroplast

Note: bp, base pair; aa, amino acid; MW, molecular weight; PI stands for Isoelectric point.

**Table 2:** Sequence characteristics and physicochemical properties of *RsCKX* genes in radish.

Gene Name	Gene ID	Chromosome	Gene Length (bp)	Protein Length (aa)	MW (Da)	PI	Predicted Localization
*RsCKX1-1*	*Rsa10025968*	R03	1709	432	48,162.5	7.75	Mitochondrion
*RsCKX1-2*	*Rsa10039146*	R01	1409	387	43,085.8	8.39	Mitochondrion
*RsCKX1-3*	*Rsa10011234*	R04	2028	440	48,832.3	9.22	Mitochondrion
*RsCKX2-1*	*Rsa10025912*	R09	4169	508	56,240.3	6.61	Plasma Membrane
*RsCKX2-2*	*Rsa10031296*	R01	8890	506	56,077	6.02	Chloroplast
*RsCKX3-1*	*Rsa10019391*	R02	2619	424	47,506.6	6.87	Extracell
*RsCKX3-2*	*Rsa10023843*	R09	2391	425	47,456.9	7.67	Vacuole
*RsCKX4*	*Rsa10036909*	R04	3204	469	51,700.7	6.6	Extracell
*RsCKX5*	*Rsa10034185*	R07	1994	407	45,073.9	6.06	Chloroplast
*RsCKX6*	*Rsa10027735*	R05	2192	430	48,062.9	8.48	Mitochondrion
*RsCKX7-1*	*Rsa10021101*	R07	3676	526	57,887.1	4.82	Cytoplasm
*RsCKX7-2*	*Rsa10013210*	R02	2058	421	45,749.1	4.38	Cytoplasm

Note: bp, base pair; aa, amino acid; MW, molecular weight; PI stands for Isoelectric point.

**Table 3:** Ka and Ks analyses of *IPT* and *CKX* genes in *Arabidopsis thaliana* and *Raphanus sativus*.

Orthologous Pairs	Ka	Ks	Ka/Ks	MYA ^a^	Paralogous Pairs	Ka	Ks	Ka/Ks	MYA ^a^
*AtIPT1/RsIPT1*	0.15	0.59	0.26	19.57	*RsIPT3-1/RsIPT3-2*	0.07	0.43	0.16	14.31
*AtIPT2/RsIPT2*	0.11	0.44	0.25	14.53	*RsIPT5-1/RsIPT5-2*	0.07	0.47	0.16	15.66
*AtIPT3/RsIPT3-1*	0.08	0.56	0.14	18.77	*RsIPT7-1/RsIPT7-2*	0.03	0.19	0.15	6.20
*AtIPT3/RsIPT3-2*	0.09	0.62	0.14	20.78	*RsIPT8-1/RsIPT8-2*	0.12	0.38	0.30	12.68
*AtIPT5/RsIPT5-1*	0.07	0.54	0.12	18.13	*RsIPT9-1/RsIPT9-2*	0.06	0.43	0.14	14.43
*AtIPT5/RsIPT5-2*	0.08	0.45	0.18	15.03	*RsCKX1-1/RsCKX1-2*	0.10	0.44	0.23	14.72
*AtIPT7/RsIPT7-1*	0.07	0.44	0.15	14.68	*RsCKX1-1/RsCKX1-3*	0.07	0.46	0.15	15.47
*AtIPT7/RsIPT7-2*	0.07	0.45	0.15	14.97	*RsCKX1-2/RsCKX1-3*	0.09	0.58	0.16	19.26
*AtIPT8/RsIPT8-1*	0.14	0.61	0.24	20.17	*RsCKX2-1/RsCKX2-2*	0.10	0.37	0.27	12.24
*AtIPT8/RsIPT8-2*	0.18	0.51	0.34	17.11	*RsCKX3-1/RsCKX3-2*	0.05	0.34	0.15	11.47
*AtIPT9/RsIPT9-1*	0.10	0.39	0.26	12.83	*RsCKX7-1/RsCKX7-2*	0.05	0.39	0.12	12.86
*AtIPT9/RsIPT9-2*	0.09	0.42	0.22	14.01					
*AtCKX1/RsCKX1-1*	0.07	0.38	0.18	12.73					
*AtCKX1/RsCKX1-2*	0.10	0.44	0.24	14.75					
*AtCKX1/RsCKX1-3*	0.07	0.46	0.15	15.43					
*AtCKX2/RsCKX2-1*	0.10	0.50	0.21	16.57					
*AtCKX2/RsCKX2-2*	0.09	0.49	0.19	16.42					
*AtCKX3/RsCKX3-1*	0.07	0.32	0.22	10.69					
*AtCKX3/RsCKX3-2*	0.07	0.37	0.18	12.44					
*AtCKX4/RsCKX4*	0.09	0.44	0.20	14.73					
*AtCKX5/RsCKX5*	0.03	0.48	0.07	16.10					
*AtCKX6/RsCKX6*	0.06	0.46	0.14	15.42					
*AtCKX7/RsCKX7-1*	0.05	0.53	0.09	17.56					
*AtCKX7/RsCKX7-2*	0.05	0.52	0.10	17.47					

Note: Ka stands for non-synonymous; Ks stands for synonymous; MYA ^a^ stands for millions of years ago.

**Table 4:** Predicted secondary structure contents of *RsCKX* proteins.

Group Type	Gene ID	Secondary Structure (%)									
		Hh	Gg	Ti	Bb	Ee	Tt	Ss	Cc	Ambiguous States	Other States
Group I	*RsCKX1-1*	33.4	0	0	0	19.72	7.19	0	39.68	0	0
	*RsCKX1-2*	28.24	0	0	0	22.28	7.25	0	42.23	0	0
	*RsCKX1-3*	30.07	0	0	0	20.5	6.15	0	43.28	0	0
	*RsCKX6*	35.2	0	0	0	18.18	5.83	0	40.79	0	0
Group II	*RsCKX2-1*	34.12	0	0	0	18.54	6.71	0	40.63	0	0
	*RsCKX2-2*	37.23	0	0	0	19.21	6.14	0	37.43	0	0
	*RsCKX3-1*	36.4	0	0	0	18.44	6.15	0	39.01	0	0
	*RsCKX3-2*	33.25	0	0	0	19.81	5.19	0	41.75	0	0
	*RsCKX4*	33.76	0	0	0	18.38	6.41	0	41.45	0	0
Group III	*RsCKX5*	33.5	0	0	0	19.21	4.93	0	42.36	0	0
Group IV	*RsCKX7-1*	32.95	0	0	0	17.9	6.29	0	42.86	0	0
	*RsCKX7-2*	31.67	0	0	0	17.38	6.43	0	44.52	0	0

Note: Hh: alpha helix; Gg: 310 helix; Ti: Pi helix; Bb: beta brige; Ee: extended strand; Tt: beta turn; Ss: beta region; Cc: random coil.

**Table 5:** RMSD values of pairwise alignment among the 3D structures of *RsCKX* proteins.

	*RsCKX1-1*	*RsCKX1-2*	*RsCKX1-3*	*RsCKX2-1*	*RsCKX2-2*	*RsCKX3-1*	*RsCKX3-2*	*RsCKX4*	*RsCKX5*	*RsCKX6*	*RsCKX7-1*	*RsCKX7-2*
*RsCKX1-1*	0	0.017	0.014	0.532	0.543	0.541	0.534	0.529	0.12	0.085	0.667	0.662
*RsCKX1-2*		0	0.023	0.459	0.457	0.472	0.477	0.493	0.129	0.095	0.519	0.509
*RsCKX1-3*			0	0.529	0.539	0.54	0.528	0.528	0.118	0.089	0.66	0.671
*RsCKX2-1*				0	0.075	0.131	0.134	0.118	0.518	0.54	0.833	0.798
*RsCKX2-2*					0	0.139	0.146	0.111	0.517	0.546	0.825	0.467
*RsCKX3-1*						0	0.079	0.135	0.526	0.536	0.813	0.817
*RsCKX3-2*							0	0.157	0.517	0.534	0.842	0.831
*RsCKX4*								0	0.524	0.529	0.835	0.774
*RsCKX5*									0	0.103	0.702	0.689
*RsCKX6*										0	0.627	0.629
*RsCKX7-1*											0	0.025
*RsCKX7-2*												0

Note: RMSD, root mean square deviation, stands for the degree to which an atom deviates from its alignment position.

## Data Availability

Data are contained within the article or Supplementary Materials.

## Supplementary Materials

The supporting information can be downloaded at: https://www.mdpi.com/article/10.3390/ijms25168974/s1.
